# G protein-coupled receptor kinase 3 modulates mesenchymal stem cell proliferation and differentiation through sphingosine-1-phosphate receptor regulation

**DOI:** 10.1186/s13287-022-02715-4

**Published:** 2022-01-29

**Authors:** Jaime M. Brozowski, Roman G. Timoshchenko, D. Stephen Serafin, Brittney Allyn, Jessica Koontz, Emily M. Rabjohns, Rishi R. Rampersad, Yinshi Ren, Amanda M. Eudy, Taylor F. Harris, David Abraham, Daniel Mattox, Clinton T. Rubin, Matthew J. Hilton, Janet Rubin, Nancy L. Allbritton, Matthew J. Billard, Teresa K. Tarrant

**Affiliations:** 1 Department of Microbiology and Immunology, University of North Carolina at Chapel Hill, Chapel Hill, NC USA; 2 Thurston Arthritis Research Center, University of North Carolina at Chapel Hill, Chapel Hill, USA; 3 Department of Medicine, Division of Rheumatology and Immunology, Duke University, 200 Trent Dr., DUMC 3874, Durham, NC USA; 4 Department of Orthopaedic Surgery, Duke Orthopaedic Cellular, Developmental and Genome Laboratories, Duke University School of Medicine, Durham, NC USA; 5 Department of Chemistry, University of North Carolina at Chapel Hill, Chapel Hill, NC USA; 6 Department of Biomedical Engineering at Stony, Brook University, Stony Brook, NY USA; 7 Department of Medicine, University of North Carolina at Chapel Hill, Chapel Hill, NC USA; 8 School of Medicine, Duke University, 152 Edwin L. Jones Building, 207 Research Drive, Durham, NC 27710 USA

**Keywords:** Bone marrow-derived mesenchymal stem cells, Mesenchymal stem cells, G protein-coupled receptors (GPCRs), G protein-coupled receptor kinase 3 (GRK3), Sphingosine-1-phosphate (S1P), Sphingosine-1-phosphate receptor (S1PR), Osteogenic differentiation, Proliferation

## Abstract

**Background:**

The bone marrow niche supports hematopoietic cell development through intimate contact with multipotent stromal mesenchymal stem cells; however, the intracellular signaling, function, and regulation of such supportive niche cells are still being defined. Our study was designed to understand how G protein receptor kinase 3 (GRK3) affects bone marrow mesenchymal stem cell function by examining primary cells from GRK3-deficient mice, which we have previously published to have a hypercellular bone marrow and leukocytosis through negative regulation of CXCL12/CXCR4 signaling.

**Methods:**

Murine GRK3-deficient bone marrow mesenchymal stromal cells were harvested and cultured to differentiate into three lineages (adipocyte, chondrocyte, and osteoblast) to confirm multipotency and compared to wild type cells. Immunoblotting, modified-TANGO experiments, and flow cytometry were used to further examine the effects of GRK3 deficiency on bone marrow mesenchymal stromal cell receptor signaling. Microcomputed tomography was used to determine trabecular and cortical bone composition of GRK3-deficient mice and standard ELISA to quantitate CXCL12 production from cellular cultures.

**Results:**

GRK3-deficient, bone marrow-derived mesenchymal stem cells exhibit enhanced and earlier osteogenic differentiation in vitro. The addition of a sphingosine kinase inhibitor abrogated the osteogenic proliferation and differentiation, suggesting that sphingosine-1-phosphate receptor signaling was a putative G protein-coupled receptor regulated by GRK3. Immunoblotting showed prolonged ERK1/2 signaling after stimulation with sphingosine-1-phosphate in GRK3-deficient cells, and modified-TANGO assays suggested the involvement of β-arrestin-2 in sphingosine-1-phosphate receptor internalization.

**Conclusions:**

Our work suggests that GRK3 regulates sphingosine-1-phosphate receptor signaling on bone marrow mesenchymal stem cells by recruiting β-arrestin to the occupied GPCR to promote internalization, and lack of such regulation affects mesenchymal stem cell functionality.

**Supplementary Information:**

The online version contains supplementary material available at 10.1186/s13287-022-02715-4.

## Introduction

Mesenchymal stem cells are multipotent stromal cells that possess the ability to differentiate into mesodermal tissues, such as chondrocytes, adipocytes, and osteocytes [1]. Mesenchymal stem cells that reside within the bone marrow microenvironment are often referred to as bone marrow *niche* mesenchymal stem cells (BmMSCs), secrete high levels of chemokine C-X-C Motif Chemokine Ligand 12 (CXCL12), which can bind to G protein-coupled receptor (GPCR) C-X-C Chemokine Receptor Type 4 (CXCR4) on hematopoietic stem and progenitor cells (HSPCs) to affect their growth and development [2, 3]. We previously published that mice deficient in *G protein-coupled receptor kinase 3* (*Grk3*), an intracellular kinase that negatively regulates GPCRs, have a hypercellular bone marrow comprised of increased Lin- Sca1 + c-Kit + (LSK) HSPCs and selective downstream committed progenitors and an increase of total white blood cells in the peripheral blood (leukocytosis) compared to wildtype (WT) mice [4]. GRK3 deficiency affects CXCR4 regulation on hematopoietic cells in mice [4] and humans [5]; however, the regulatory effects of GRK3 on BmMSC function and BmMSC GPCRs are not well defined. Due to the importance of BmMSCs on HSPC biology, we wished to better clarify how GRK3, which is expressed in BmMSCs [6], affects their function and through which GPCR these effects may be regulated.

We observed *Grk3-/-* BmMSCs have enhanced osteogenic differentiation, higher levels of CXCL12, and increased proliferation. Although we have previously published that global deficiency of GRK3 leads to bone marrow hypercellularity and leukocytosis in mice [4], microcomputed tomography (µCT) of cortical and trabecular bone did not show substantial clinical differences in bone density measurements. Accumulating evidence supports that phospholipid sphingosine-1-phosphate (S1P) affects mesenchymal stem cell function [7] in osteogenic differentiation [8–10] and proliferation [11, 12]; thus, we examined GRK3-dependent S1PR antagonism, signaling, and β-arrestin-2 recruitment to determine the interplay between these protein interactions in BmMSC biology. Establishing functional relationships in chemokine receptor signaling of stem cells relevant to the bone marrow niche may further facilitate mechanistic understanding of stem cell biology and potential therapeutic targeting of stem cells for the treatment of cancer, autoimmunity, and immunodeficiency disorders.

## Materials and methods

### Animals

C57BL/6 WT and *Grk3-/-* age-matched (8–12-week-old) mice were used under standard IACUC-approved protocols in the AAALAC-accredited vivarium of UNC, and care of animals was in accordance with institutional guidelines. The *Grk3-/-* mouse strain was provided by Dr. Robert J. Lefkowitz (Duke University) and backcrossed > 12 generations on the C57BL/6 background. The line is re-derived every 1–2 years to prevent genetic drift from the C57BL/6 strain.

### Bone marrow-derived mesenchymal stem cell isolations

BmMSC were isolated from RPMI-flushed femurs and tibias of two male mice and isolated in complete isolation media (CIM) containing RPMI (Corning, 10-040-CV) with 10% fetal bovine serum (FBS, Atlanta Biologicals, S12450), 10% horse serum (HS, HyClone, SH30074.03), 1% 100 IU/mL penicillin G/100 µg/mL streptomycin (P/S, Corning, 30-002-Cl), and 12 µM final concentration of L-Glutamine (Corning, 25-005-Cl), as similar methodologies have been previously described [13–15]. Once BmMSC were isolated, the cells were plated and cultured for expansion in complete expansion media (CEM) containing IMDM (Gibco, 12440–053), 10% FBS, 10% HS, 1% P/S, and 12 µM final concentration of L-Glutamine [14, 15], which was followed by hematopoietic CD45 (Stem Cell Technologies, 19771) and CD11b (Miltenyi, 130-049-601 or 130-093-634) depletion, as recommended by [13], via magnetic negative selection at early passages 1–3. BmMSCs were passaged at 70–80% confluency. The majority of experiments were performed at passages 10–13 and are specified throughout the manuscript.

### Chondrogenic, adipogenic, and osteogenic differentiation

*Chondrogenic differentiation.* BmMSCs were suspended in CEM at 1.6 × 10^7^ viable cells/mL. Micromasses were made by adding 5 µL droplets of the cell suspension onto a 6-well plate and given 3–4 h to attach. Chondrogenic media (Gibco StemPro® Chondrogenesis Differentiation Kit, A10071-01) supplemented with penicillin streptomycin was added to each well and incubated for 21 days. Cells were fixed with 10% formalin and stained using Alcian Blue. *Adipogenic differentiation.* BmMSCs were plated at 1 × 10^5^ cells/well of a 6-well plate in CEM supplemented with 50 µM indomethacin, 5 µg/mL insulin, and 0.1 µM dexamethasone. Cells were fixed with 10% formalin and stained using Oil Red O. *Osteogenic differentiation.* BmMSCs were plated at 1 × 10^5^ cells/well of a 6-well plate in CEM supplemented with 50 µg/mL ascorbic acid and 20 mM ß-glycerophosphate. Cells were fixed with 10% formalin and stained using Alizarin Red. For sphingosine kinase inhibitor (SKI)-treated osteogenic differentiation, BmMSCs were plated at 2 × 10^4^ cells/well in a 24-well plate in CEM, and osteogenic differentiation was induced after an overnight rest. Cells were treated with sphingosine kinase inhibitor 2 (SKI, Cayman Chemical, 10009222) at a final concentration of 5 µM or vehicle (DMSO). Fresh media changes occurred every third day using CEM plus SKI or vehicle. Cells were fixed with 10% formalin and stained using Alizarin Red stain for analysis. Images were captured using the Olympus 1X-81 microscope and MetaMorph software.

### Bone marker mRNA expression (qRT-PCR)

Total RNA from BmMSCs undergoing osteogenic differentiation was prepared using the RNeasy Mini/ Plus kit (Qiagen) according to manufacturer’s instructions. Reverse transcriptase cDNA synthesis was performed using iScript cDNA synthesis kit (Bio-Rad, 170-8891). qRT-PCR was performed in duplicate (SYBR® Green, Bio-Rad, 172-5261) and normalized to housekeeping gene IDUA. Mean fold change of the following markers listed by primers below were determined by -2ΔΔCt with WT day 0 as control. Primers utilized for qRT-PCR were Alkaline Phosphatase forward: AAG GCT TCT TCT TGC TGG TG, Alkaline Phosphatase reverse: GCC TTA CCC TCA TGA TGT CC; Osteocalcin forward: CTG ACC TCA CAG ATC CCA AGC, Osteocalcin reverse: TGG TCT GAT AGC TCG TCA CAA G; Osteopontin forward: AGC AAG AAA CTC TTC CAA GCA A, Osteopontin reverse: GTG AGA TTC GTC AGA TTC ATC CG; Osteonectin forward: GTG GAA ATG GGA GAA TTT GAG GA, Osteonectin reverse: CTC ACA CAC CTT GCC ATG TTT; Osterix forward: AGC GAC CAC TTG AGC AAA CAT, Osterix reverse: GCG GCT GAT TGG CTT CTT CT; IDUA forward: GCA TCC AAG TGG GTG AAG TT and IDUA reverse: CAT TGA GCA GGT CCG GAT AC.

### ELISA

BmMSCs were plated at 1 × 10^5^ cells/well of a 6-well plate in CEM and rested overnight for attachment before supernatant collections began at baseline (day 1) and each subsequent collection. Supernatants of undifferentiated BmMSC monolayers were collected and analyzed for CXCL12 protein using the CXCL12 DuoSet ELISA kit (R&D Systems, DY460), as per instructions.

### Microcomputed tomography (µCT)

For 8–12-week-old mice, µCT imaging was used to analyze the trabecular bone morphology at the distal femur at 12 micron resolution. The metaphyseal region of the distal femur was scanned beginning 720 microns proximal to the growth plate and extending 1500 microns toward the diaphysis of the femur. An automatic script was used to analyze the region of interest to separate the trabecular and cortical regions of the bone and quantify bone morphology. Trabecular analysis includes quantification of BV/TV (bone volume/total volume). For 17–20-month-old aged mice, µCT imaging morphology (VivaCT80, Scanco Medical, Basserdorf, Switzerland) was used to analyze the trabecular bone. Briefly, metaphysis region was selected for 100 slices under the femur growth plate. Trabecular analysis includes quantification of BV/TV. Analyses were conducted at 12 μm slice increment with an integration time of 300 ms, a current of 145 mA, and an energy setting of 55 kV. The threshold was chosen using 2D evaluation of several slices in the transverse anatomic plane so that mineralized bone was identified but surrounding soft tissue was excluded.

### Cellular proliferation

Proliferation of 5 × 10^4^ BmMSCs/well of a 6-well plate was analyzed after exogenous CCK-8 (Dojindo Molecular Technologies) was added to each well at the indicated timepoints. Absorbance was measured after 3 h incubation using the Promega Glomax® Multi + Detection System. Data were analyzed by deducting background (media and CCK-8) absorbance from raw absorbance reads. For S1P studies, BmMSCs were plated at 1 × 10^4^ cells/well in a 24-well plate and treated with SKI at a final concentration of 5 µM.

### CXCL12 per cell measurement

To quantify CXCL12 production per BmMSC, cells were plated at 9,374 cells/cm2 and cultured for 5 days in CEM. On day 5, supernatants were collected, cells were washed with PBS prior to trypsinization, and live cells were counted using trypan blue exclusion. A CXCL12 ELISA (Sigma, St. Louis, MO, USA) was used to determine the concentration of CXCL12 in the culture supernatant, which was then divided by the number of cells in that well to obtain the amount of CXCL12 secreted per cell.

### Immunoblotting

BmMSCs were plated in CEM at 2.25 × 10^5^ cell density in a 6-well plate and incubated overnight. DPBS rinsed cells were rinsed three times with IMDM containing 20% charcoal-stripped FBS (to remove serum S1P) and 1% P/S and incubated at 37 °C for 15 min. Fresh media was added, and BmMSCs were stimulated with 1 µM S1P for indicated timepoints. Unstimulated BmMSCs served as 0 min timepoint control. Following stimulation with S1P, BmMSC cells were rinsed with DPBS and lysed in cold HBSS + 1% TritonX100 lysis buffer containing protease inhibitors (1 mM PMSF, 1 µg/mL aprotinin, 1 µg/mL pepstatin, and 1 µg/mL leupeptin) and phosphatase inhibitors (5 mM NaF and 2 mM NaVO_4_). All WT and *Grk3-/-* BmMSC lysates were normalized via bicinchoninic acid (BCA) assay, and 6 µg of total protein in Laemmli sample buffer (non-reducing) was freshly loaded onto AnyKD Mini-PROTEAN®TGX precast protein gel (Bio-Rad, 4569036). Gels were run at 100 V for 1.5–2 h in 1X Tris/Glycine SDS buffer. Proteins were transferred overnight at 4 °C onto nitrocellulose membrane in Tris base (25 mM)/Glycine (192 mM) transfer buffer containing 20% methanol. The membrane was blocked in 3% fatty-acid free BSA in TBS plus 0.1% Tween-20 (TBS/T) for 2 h at 25 °C and incubated with primary antibody 1:2,000 phospho-p44/42 MAPK or 1:2,000–3,000 p44/42 MAPK (Cell Signaling Technologies, 4370/4695) overnight at 4 °C, or 1:10,000 GAPDH (Trevigen, 2275-PC-100) for 2 h at 4 °C. The membrane was washed three times for 10 min in TBS/T, incubated with secondary antibody 1:5,000 anti-rabbit IgG HRP (Cell Signaling Technologies, 7074) for 1 h at 25 °C, and washed twice for 10 min in TBS/T and once in TBS. Detection was performed via ECL Prime or ECL Select (GE Healthcare, RPN2232/ RPN2235) and imaged on GeneSys image acquisition software. Densitometry was obtained by measuring ratio of phospho-ERK (pERK) over total ERK using ImageJ software.

### β-arrestin recruitment assay

GRK recruitment of β-arrestin-2 to the S1PR1 carboxy-terminus was measured using agonist-stimulation in a modified-TANGO assay, as previously reported [16]. HTLA cells were transfected with a S1PR1-TCS-tTA receptor construct after removing the V2 vasopressin sequence to prevent nonspecific β-arrestin recruitment to the wildtype S1PR1. GRK overexpression was achieved via plasmids: GRK2 pcMyc_LIC and GRK3 pcMyc_LIC, and utilized a separate expression vector encoding yellow fluorescent protein (YFP) that was simultaneously transfected to serve as a transfection control. HTLA cells were transiently transfected with 6.5 μg of total plasmid DNA (3 μg of S1P-Tango, 0.5 μg of YFP, and either 3 μg of empty-vector control, GRK2, or GRK3) via calcium-phosphate precipitation. Transfection efficiency was determined by YFP epifluorescence to be consistently > 70% at 24 h post-transfection. Cells were serum starved and then stimulated with S1P ligand at varied concentrations up to 1 µM. BriteGlo reagent (Promega, Madison, WI, USA) was added for luminescence detection via Promega Glomax® Multi + Detection System (0.5 s/well). Raw data were normalized by subtracting background for each independent run and setting the lowest concentration of the control condition at 0% and highest concentration at 100%.

### S1PR1 internalization assay

BmMSCs cultured in CEM were rinsed with DPBS and incubated in serum-free CEM (SFM) for 2.5 h. BmMSCs were detached using Accutase® (Sigma, A6964), rinsed once with cold SFM containing 5% charcoal-stripped FBS (to remove serum S1P), and resuspended in cold DPBS. 1 × 10^5^ BmMSCs were stimulated with 1 µM S1P ligand in 100 µL FACS buffer (DPBS1X + 0.2% fatty acid free BSA + 0.1% sodium azide) at specific timepoints. Unstimulated BmMSCs served as 0 min timepoint control. S1PR1 internalization was halted with 2 mL of ice cold FACS buffer and sample tubes were placed on ice. BmMSCs were stained for Sca1 (eBioscience clone: D7, APC-conjugated) and S1PR1 (R&D Systems clone: 713,412, PE-conjugated) for 30 min on ice in 100 µL FACS Buffer, rinsed, and analyzed by flow cytometry.

### Statistical analyses

All data were graphed utilizing GraphPad Prism v.7 and statistically evaluated using GraphPad Prism v.7 or Microsoft Office Excel program. Taking into consideration time and strain (WT and *Grk3-/-*), the bone marker qRT-PCR, CCK-8 proliferation, and S1PR1 internalization was statistically analyzed using a RM two-way ANOVA with Sidak’s multiple comparison test, a method preferred over Bonferroni due to increased power [17, 18]. Similarly, taking into consideration time and strain (WT and *Grk3-/-*) but with multiple treatment groups, the SKI-treated CCK-8 proliferation was analyzed using a RM two-way ANOVA with Tukey’s multiple comparison test for pairwise comparisons [17, 19]. Student’s t-test compared two independent groups (WT and *Grk3-/-*) for the CXCL12-detection ELISA, microcomputed tomography data, qRT-PCR of bone markers, and western blot densitometry. Taking into consideration three independent groups (empty vector, GRK2, and GRK3), the β-arrestin recruitment assay was analyzed by one-way ANOVA with Dunnett’s multiple comparison test, which compared each group to the control (empty vector) [17, 20].

## Results

### GRK3-deficient BmMSCs have enhanced osteogenic differentiation

**Fig. 1:**
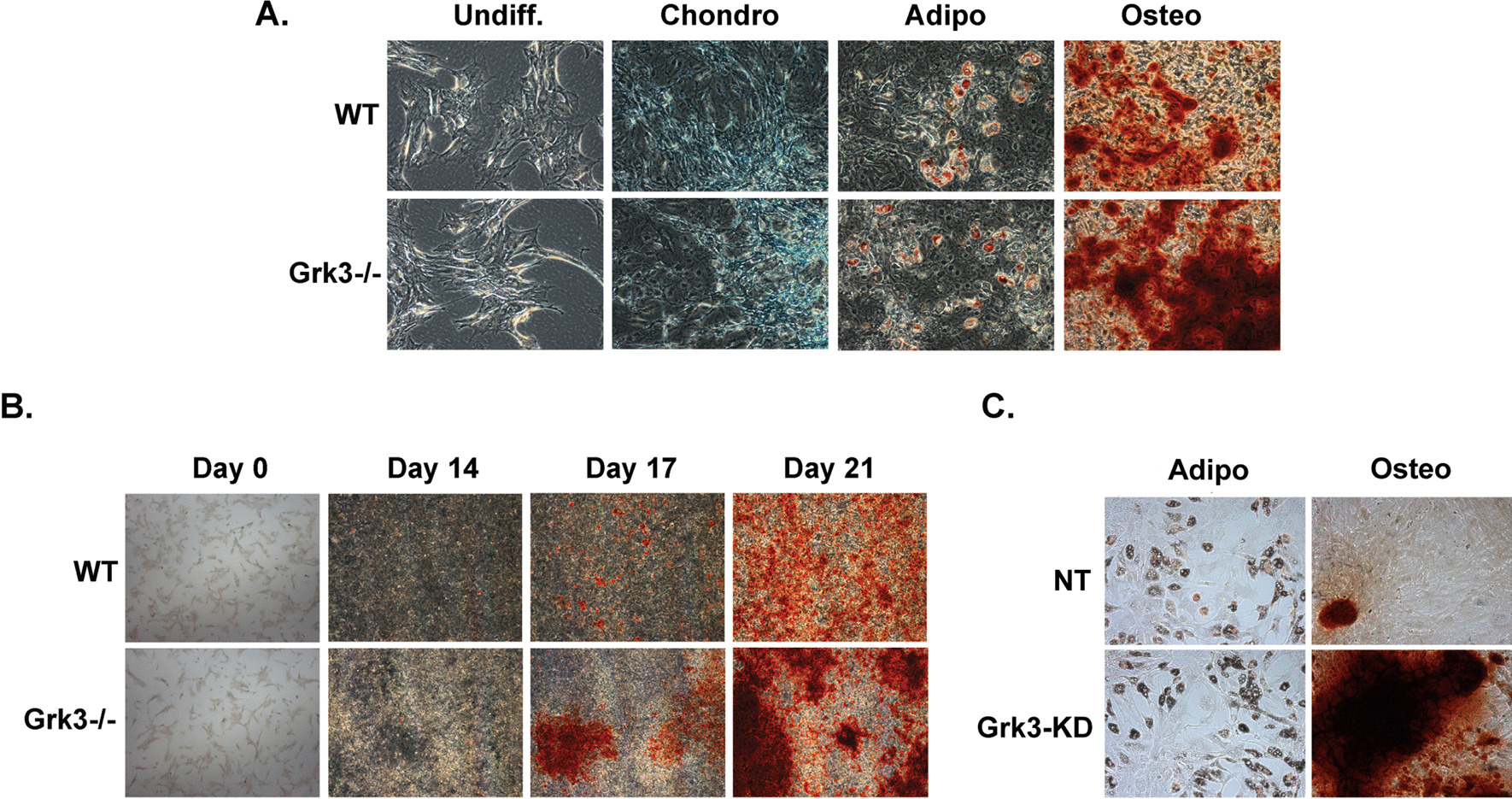
Bone marrow-derived mesenchymal stem cells (BmMSCs) deficient in G protein-coupled receptor kinase 3 (*Grk3-/-*) and *Grk3*-deficient shRNA knockdown BmMSCs have enhanced osteogenic differentiation in comparison with wildtype (WT) or nontarget knockdown (NT) control BmMSCs. (A) Multipotent differentiation of BmMSCs isolated ex vivo from *Grk3-/-* and WT mice into chondrocytes (Alcian Blue stain), adipocytes (Oil Red O stain), and osteoblasts (Alizarin Red stain) at day 21; images acquired at 10X magnification. Differentiation analyses *n* = 3 chondrogenic/adipogenic and *n* = 10 osteogenic, passages 7–11. (**B**) Time-course *Grk3-/-* and WT BmMSC osteogenic differentiation (Alizarin Red stain) at 2X magnification. Representative images, *n* = 4, passages 10 and 11. (**C**) shRNA Nontarget (NT) control and GRK3-knockdown (GRK3-KD) BmMSCs have comparable adipogenic differentiation (Oil Red O stain); however, GRK3-KD BmMSCs show enhanced osteogenic differentiation (Alizarin Red stain), similar to BmMSCs isolated from *Grk3-/-* mice. Images captured at 10X magnification using the Olympus 1X-81 microscope and MetaMorph software

**Fig. 2:**
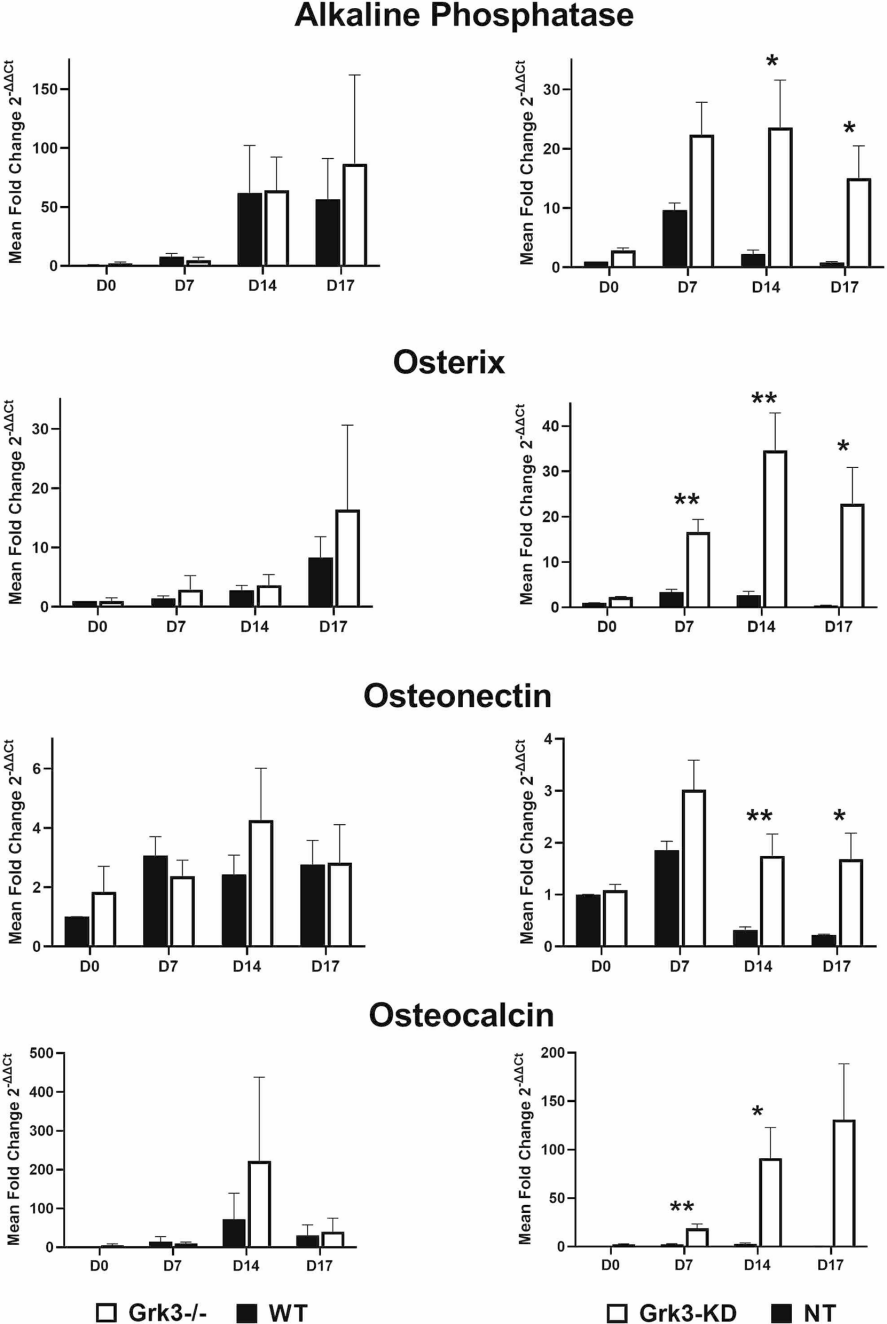
Osteogenic expression markers increase in MSC cultures during osteogenic differentiation relative to housekeeping gene IDUA. qRT-PCR of osteogenic differentiation markers alkaline phosphatase, osterix, osteonectin, and osteocalcin from BmMSC ex vivo cultures in osteogenic differentiation media over time (days 0–17). Left panels are comparing *Grk3-/-* to WT BmMSC cultures (*n* = 6, passages 10–15), and right panels are comparing *Grk3*-shRNA knockdown to nontarget shRNA control BmMSC cultures (*n* = 4, passages 11,12). * *P* ≤ 0.05 ** *P* ≤ 0.01

In our previous publication, we observed *Grk3-/-* mice had increased HSPC numbers compared to WT mice [4]. Since it is well established that bone marrow stromal cells enhance hematopoiesis [21–26], we aimed to test whether there were differences between *Grk3-/-* and WT murine stromal BmMSCs that could contribute to this phenotype. BmMSCs isolated from WT and *Grk3-/-* mice adhered to culture plastic, underwent differentiation into tissue-specific lineages to ensure multipotency (Fig. 1A), and lacked expression of CD45 and CD11b (hematopoietic cell markers) and expressed mouse mesenchymal stem cell markers Sca1, CD106, CD73, CD44, CD29 (Additional File 1: Fig. S1 and Additional File 2: S2). During differentiation analysis, we observed no substantial differences between WT and *Grk3-/-* BmMSC chondrogenic or adipogenic differentiation cultures. However, we observed enhanced and earlier osteogenic differentiation in *Grk3-/-* BmMSC cultures in comparison with WT cultures, as demonstrated by positive Alizarin Red stain (Fig. 1B) and higher mRNA expression levels of several markers of osteoblast differentiation over time including alkaline phosphatase, osterix, osteocalcin, and osteonectin (Fig. 2) [27, 28]. We noticed the enhanced osteogenic differentiation phenotype was reproducible in four separate isolations. To further ensure there was no isolation differences between WT and *Grk3-/-* BmMSCs that may induce such a phenotype, we utilized shRNA to knockdown GRK3 (*Grk3*-KD) from WT BmMSCs and induced multipotent differentiation, which again showed the identical phenotype of enhanced osteogenic differentiation in *Grk3*-KD BmMSCs in comparison with nontarget (NT) control BmMSCs (Fig. 1C). Osteoblast differentiation marker increases were statistically significant for alkaline phosphatase, osterix, osteocalcin, and osteonectin (Fig. 2). Osteopontin expression levels were detected but not found to be different between *Grk3*-deficient cultures that were isolated from *Grk3-/-* mice or as shRNA knockdowns of MSC cultures when compared to controls (data not shown).

### GRK3-deficient BmMSC cultures have higher levels of CXCL12 but there is not increased CXCL12 secretion per cell

**Fig. 3:**
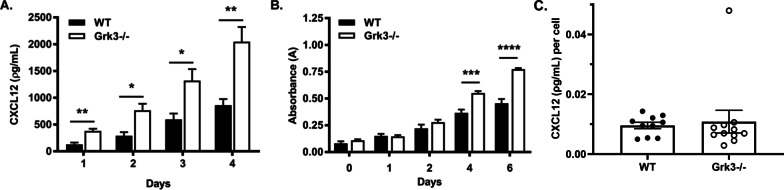
*Grk3-/-* BmMSCs have higher levels of CXCL12 detected in culture and proliferate more in comparison with WT BmMSCs but secrete similar amounts of CXCL12 per cell. (**A**) Quantification of CXCL12 protein concentration from supernatant of BmMSC cultures. Lower limit of detection at 46.9 ρg/mL. Data represent mean ± SEM, *n* = 4, passages 11 and 12. (**B**) BmMSC proliferation determined by increased formazan dye production from viable cells over time using CCK-8 proliferation assay. Data represent mean ± SEM, *n* = 3, except day 0 *n* = 2, passage 7. * *P* ≤ 0.05 ** *P* ≤ 0.01 *** *P* ≤ 0.001, **** *P* ≤ 0.0001 (**C**) CXCL12 per MSC determined by trypsinized cell count using trypan blue exclusion of individual wells divided by the amount of CXCL12 (pg/ml) measured by ELISA. Data represent mean ± SEM, *n* = 11 Grk3-/-, *n* = 10 WT, passages 10–13

BmMSCs, CXCL12-abundant reticular cells, and pre-osteoblasts isolated from bone marrow secrete CXCL12 [22, 23], an essential chemokine for HSPC development and/or function. Since we published that *Grk3-/-* mice had increased HSPCs in vivo [4], we tested whether or not there may be differences in HSPC growth factor CXCL12 between WT and *Grk3-/-* BmMSCs ex vivo*.* Our data reveal that *Grk3-/-* BmMSC cultures have higher CXCL12 levels at baseline and after subsequent days in culture in comparison with WT BmMSC (Fig. 3A). However, when CXCL12 was analyzed as a per cell calculation (Fig. 3C), CXCL12 secretion was found to be similar between WT and *Grk3-/-* MSCs.

### GRK3 deficiency increases BmMSC proliferation ex vivo

Due to the enhanced detection of CXCL12 in *Grk3-/-* BmMSC culture but a CXCL12/cell ratio that was similar between WT and *Grk3-/-* MSCs, we wanted to further investigate whether this was the result of enhanced *Grk3-/-* MSC proliferation. Cell counting kit-8 (CCK-8) quantitates proliferation by absorbance detection of increased formazan dye production from viable cells. Using this assay of cellular proliferation, we show that *Grk3-/-* BmMSCs have more proliferation in comparison with WT BmMSCs (Fig. 3B).

### GRK3 deficiency does not affect mature bone formation in vivo

**Fig. 4:**
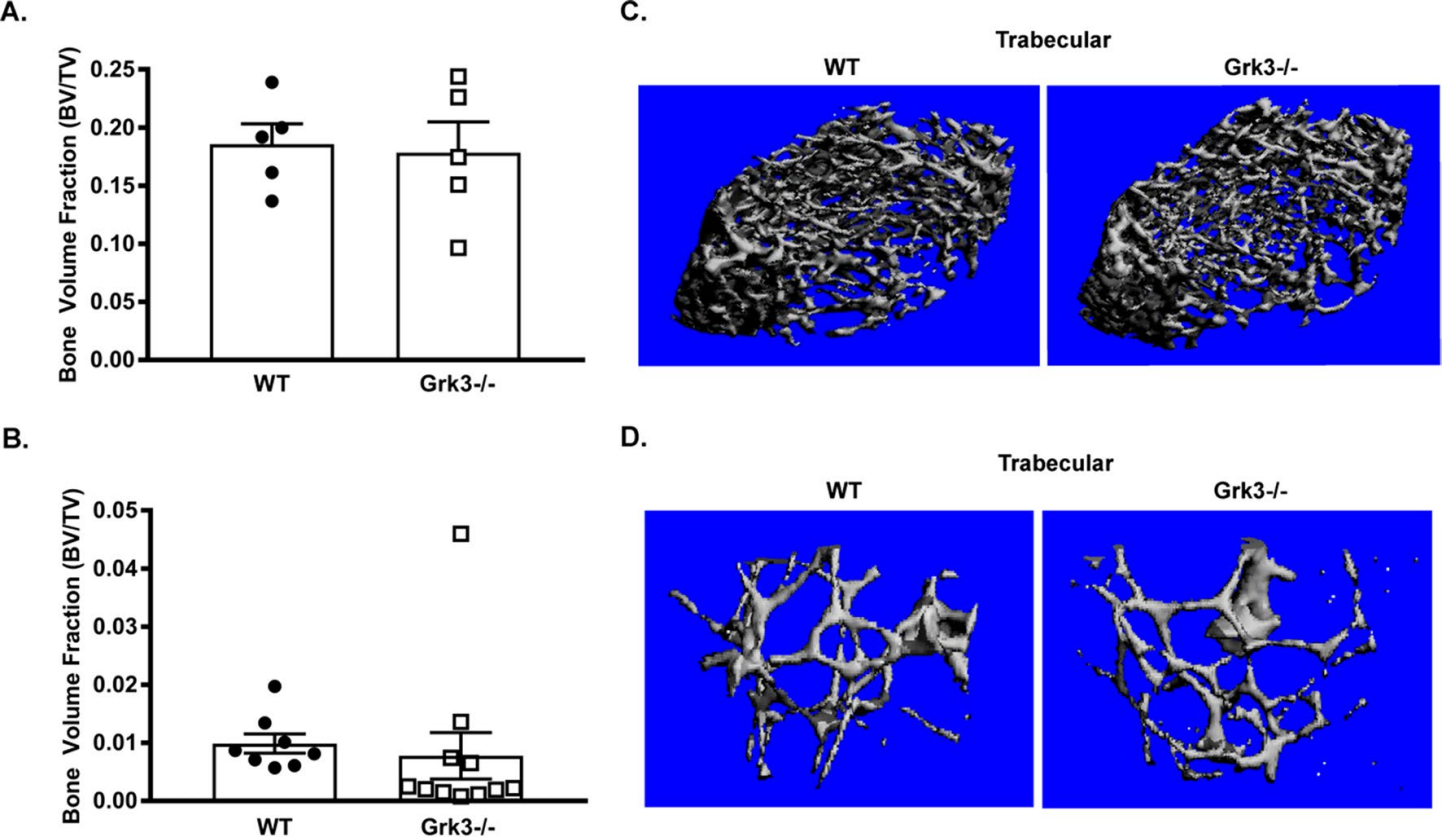
Femoral trabecular bone density is not differentially affected by GRK3 deficiency in young or aged mice. Quantification of trabecular bone volume fraction (BV/TV) via microcomputed tomography analysis of (**A**) young and (**B**) aged *Grk3-/-* and WT mouse femurs. Representative 3D image of trabecular bone of (**C**) young and (**D**) aged *Grk3-/-* and WT mouse femurs. Data represent mean ± SEM, *n* = 5 for young mice and *n* = 8 (WT) *n* = 11 (*Grk3-/-*) for aged female mice

Because *Grk3-/-* BmMSC osteogenic differentiation and proliferation are increased ex vivo, we investigated whether or not GRK3 deficiency affected mature bone development and susceptibility to osteoporosis in vivo. We performed µCT femoral bone analyses on WT and *Grk3-/-* young (8–12 weeks) and aged (17–20 months) female mice. No statistical differences in the trabecular bone volume fraction of *Grk3-/-* young or aged female mice were observed between genotypes (Fig. 4A–D).

### Inhibition of S1P reduces the enhanced osteogenic differentiation and proliferation phenotype of *Grk3-/-* BmMSCs

Since GRK3 regulates signaling of cell-surface expressed GPCRs [6], we next aimed to identify the putative GPCR that could be contributing to the enhanced *Grk3-/-* BmMSC phenotypes of osteogenic differentiation and proliferation. Since we and others have demonstrated that BmMSCs secrete CXCL12 [22, 23], that GRK3 regulates CXCR4 in other cell types [4, 5, 29], and that GRK3 regulation of CXCR4 impacts bone marrow hematopoietic cell functionality [4, 30], we hypothesized CXCL12 may be acting via an autocrine CXCL12/CXCR4 signaling loop on BmMSCs. However, neither agonist stimulation nor antagonist inhibition of CXCR4 signaling affected the increased *Grk3-/-* BmMSCs proliferation that we observed (Additional File 3: Fig. S3).

**Fig. 5:**
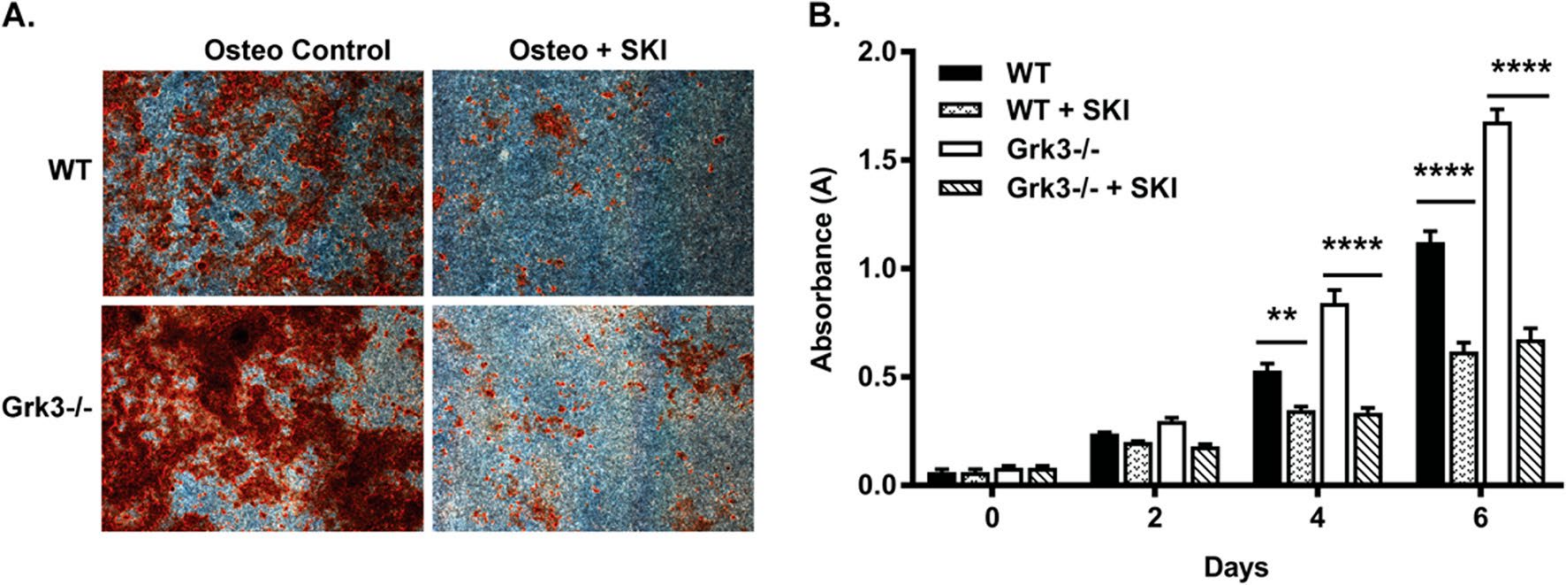
Osteogenic differentiation and proliferation of *Grk3-/-* BmMSCs are decreased to WT levels with sphingosine kinase inhibitor treatment. (**A**) BmMSCs treated with 5 µM SKI while in osteogenic media for differentiation studies and stained with Alizarin Red. Representative images acquired at 2X magnification, *n* = 3, 13, 14. (**B**) BmMSCs cultured with 5 µM SKI in culture media for proliferation detection over time. Data represent mean ± SEM, *n* = 3, passage 11 **P ≤ 0.01 ****P ≤ 0.0001

Accumulating evidence supports phospholipid S1P affects mesenchymal stem cell function [7], specifically in osteogenic differentiation [8–10] and proliferation [11, 12]. Since S1P binds to GPCR S1P receptors (S1PR), we next hypothesized that GRK3 was negatively regulating S1PR signaling, leading to functional outcomes of enhanced BmMSC proliferation and osteogenic differentiation. To test this hypothesis, we had to address that S1P is readily available in the BmMSC culture media due to the naturally high presence in fetal bovine and horse serum [31] and that murine BmMSCs secrete S1P [32], similarly to CXCL12. Therefore, to effectively neutralize S1P in culture, we utilized a sphingosine kinase inhibitor (SKI), which reduces the conversion of sphingosine to active ligand S1P. We also verified that WT and *Grk3-/-* BmMSCs had comparable sphingosine kinase activity before manipulation of this pathway (Additional File 4: Fig. S4). Titration of SKI to an optimized dose of 5 µM reduced both *Grk3-/-* osteogenic differentiation (Fig. 5A) and proliferation (Fig. 5B) to comparable levels with WT BmMSCs, suggesting S1PR, and not CXCR4, has a role in the enhanced phenotypes.

### *Grk3-/-* BmMSCs have enhanced ERK1/2 signaling after S1P stimulation

**Fig. 6:**
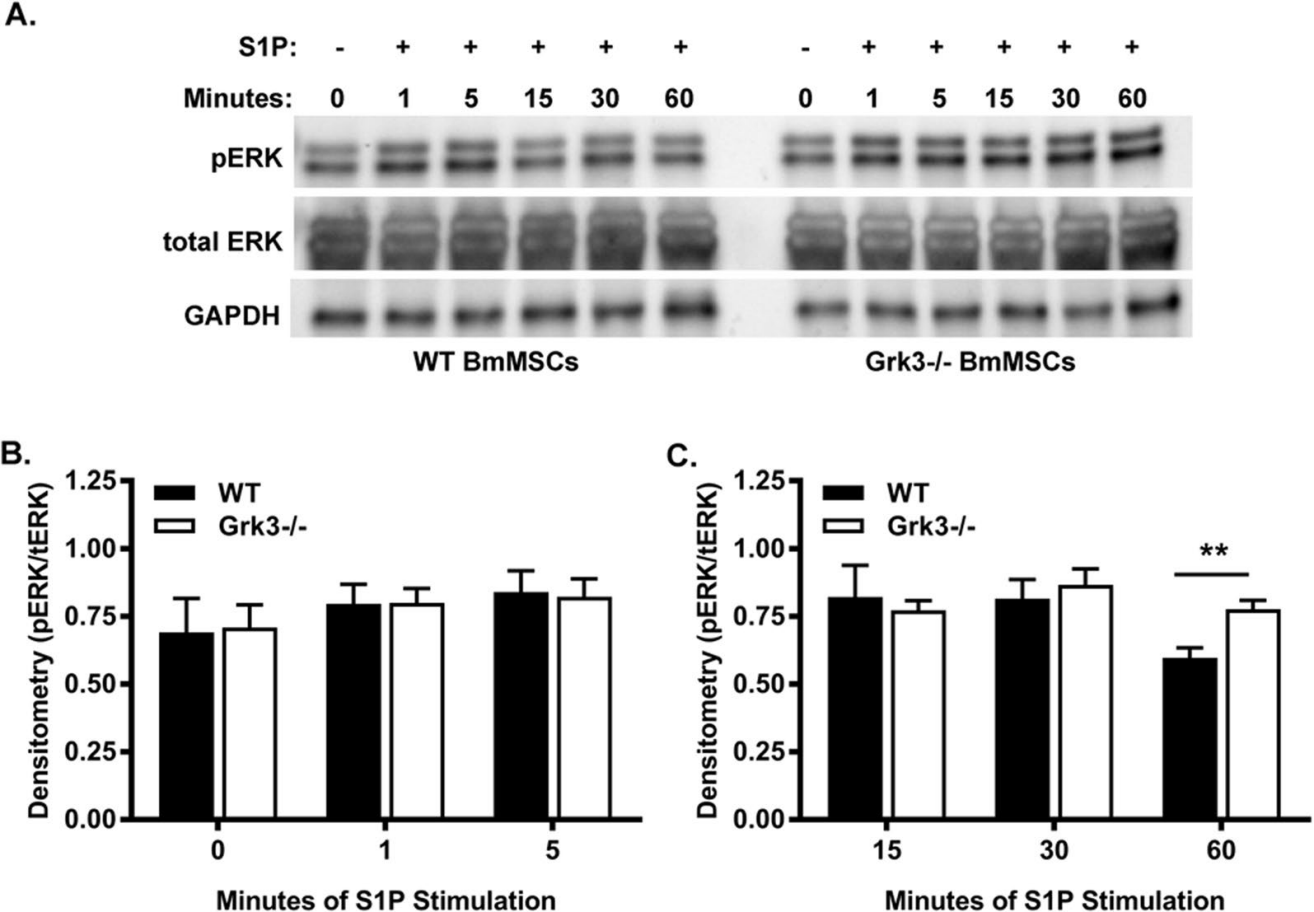
*Grk3-/-* BmMSCs have enhanced ERK1/2 signaling after S1P stimulation at later timepoints. (**A**) WT (left) and *Grk3-/-* (right) BmMSCs were stimulated with 1 µM S1P for designated times for analysis of ERK1/2 signaling; phospho-ERK (pERK) and total ERK (tERK). Representative blot, *n* = 4. The pERK/tERK ratio for each blot was quantified via densitometry for (**B**) early-stage ERK signaling at 0, 1, and 5 min and (**C**) late-stage ERK signaling at 15, 30, and 60 min. Data represent mean ± SEM, *n* = 4, passages 13 and 15. ** *P* ≤ 0.01

The extracellular-related kinase (ERK) signaling pathway has been linked to both mesenchymal stem cell [33] and pre-osteoblastic cell line [34] osteogenic differentiation, as well as mesenchymal stem cell proliferation [12]; therefore, we hypothesized ERK signaling may be enhanced in *Grk3-/-* BmMSCs with S1P stimulation. We show via densitometry ERK1/2 early signaling at 0, 1, and 5 min is comparable between WT and *Grk3-/-* BmMSCs (Fig. 6A, B); however, during late signaling, while WT signaling wanes after 30 min, *Grk3-/-* BmMSCs show sustained signaling still at 60 min (Fig. 6A, C).

### GRK3 recruits β-arrestin to the C-terminus of S1PR1 and affects S1PR1 internalization

While BmMSCs express S1PRs 1-3 [35–37], the intracellular signaling, function, and regulation of each specific receptor on BmMSCs is still being defined. Recent studies have shown stimulatory BmMSC function through ligation of S1PR1 and S1PR3 in contrast to inhibition of function after stimulation through S1PR2 [36]. We hypothesized our enhanced GRK3-regulatory phenotype may be mediated via S1PR1, since our *Grk3-/-* BmMSCs phenotype showed enhanced proliferation and prolonged ERK1/2 signal activation, analogous to previously reported S1PR1 activation in adipose-derived mesenchymal stem cells [12]. S1PR1 was also clearly detected on WT and *Grk3-/-* BmMSCs by protein lysate analysis and flow cytometry (Additional File 5: Fig. S5A, B).

**Fig. 7:**
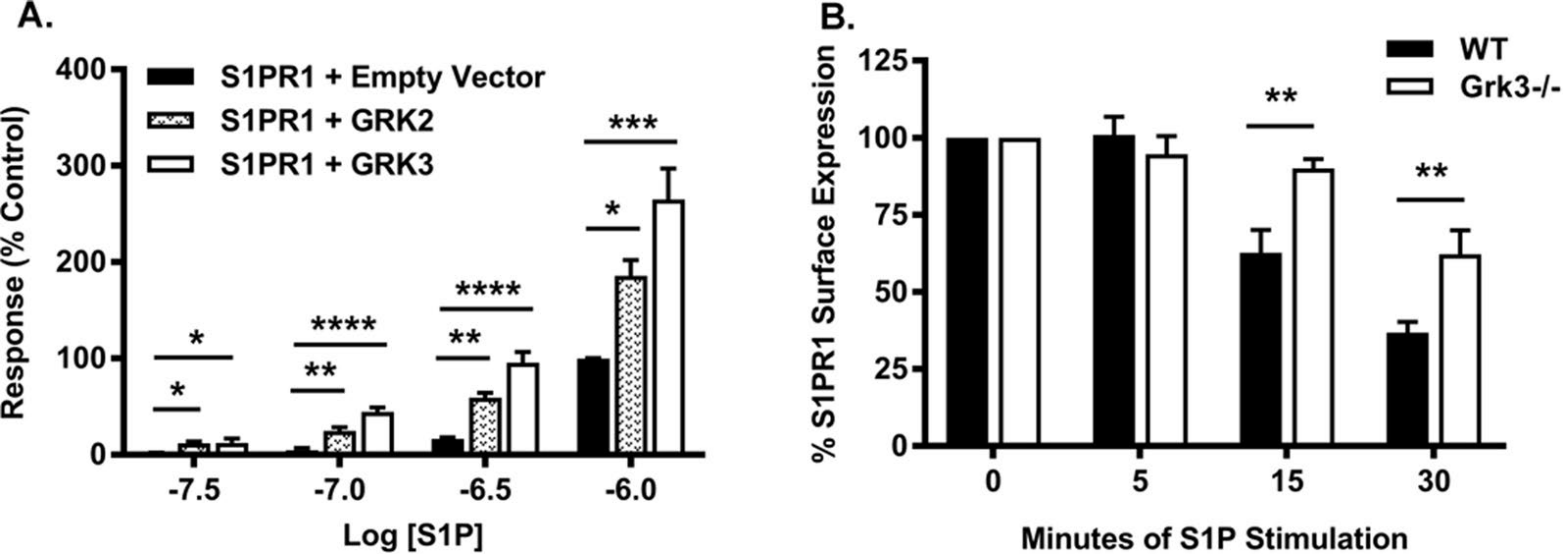
GRK3 recruits β-arrestin to the C-terminus of S1PR1 and affects S1PR1 internalization. (**A**) HTLA cells transfected with S1PR1-TCS-tTA receptor construct and empty vector (negative control), GRK2 (positive control), or GRK3 plasmids and stimulated with 1 µM S1P for detection of β-arrestin recruitment response via luminescence. Data represent mean ± SEM, *n* = 6. (**B**) Sphingosine-1-phosphate receptor 1 (S1PR1) surface expression as measured by median fluorescence intensity compared to unstimulated cells (0 min) by flow cytometry. WT and *Grk3-/-* BmMSCs were stimulated with 1 µM S1P at designated timepoints. Data represent mean ± SEM, *n* = 5, passages 11, 12, 13. *P ≤ 0.05 **P ≤ 0.01 ***P ≤ 0.001 ****P ≤ 0.0001

We hypothesized that GRK3 could be involved in S1PR1 β-arrestin recruitment—a protein necessary for GPCR internalization and another pathway of GPCR regulation in addition to receptor desensitization. Using the modified-TANGO assay [16, 38] with GRK2 as a positive control [39], we demonstrated GRK3 recruits β-arrestin to the C-terminus of S1PR1 (Fig. 7A). We next hypothesized that receptor internalization was an additional means of GRK3/S1PR1 regulation, and thus S1PR1 internalization would be impaired in *Grk3-/-* BmMSCs. In S1P-activated receptor internalization assays, S1PR1 surface receptor expression by flow cytometry was prolonged over time in *Grk3-/-* BmMSCs compared to controls (Fig. 7B), suggesting a defect in β-arrestin-mediated internalization.

## Discussion

Mesenchymal stem cells are under active investigation for use in regenerative medicine and cell therapy; therefore, there is an essential need for better cellular characterization and understanding of their signaling, function, and regulation [40–42]. Challenges within the field include ex vivo expansion studies utilizing different tissue-derived sources of cells, growth media conditions, serum sources and lots, seeding densities, and sample variations [40, 43], which can be potential confounders in interpreting data. To reduce such confounders, we utilized media previously published to best support BmMSCs from C57BL/6 mice [14], maintained the same serum sources and lots throughout all studies, seeded and cultured cells at comparable densities, and minimized sample-variation findings by design and testing of several different BmMSC isolations from WT and *Grk3-/-* mice. We also performed the majority of experiments at passages 10–13 and have specified specific passages used for experimentation in figure legends.

Our WT and *Grk3-/-* BmMSC cultures had similar levels of mesenchymal stem cell markers Sca1, CD106, CD44, CD29 (Additional File 1: Fig. S1 and Additional File 2: S2); however, there was some variability of CD73, a marker of multipotency [44], in some batches. The variability was more pronounced in wild type control cultures, albeit this was not statistically significant (Additional File 2: Fig. S2). The variability does highlight the challenge when working with separate BmMSC isolates that can lead to sample variation [40, 43]. Given that the CD73 expression was variable but the phenotypes remained intact between *Grk3-/-* and WT MSCs batches, we hypothesize that CD73 does not explain the differences we observe between the genotypes in osteogenic differentiation and proliferation.

We observe in Fig. 2 that osteogenic markers were increased over time in both *Grk3-/-* BmMSC cultures isolated from *Grk3-/-* mice and in *Grk3*-deficient shRNA knockdown BmMSCs when compared to control cultures and that this correlates with the earlier and enhanced morphologic osteogenic differentiation observed in culture (Fig. 1). Expression data from the *Grk3-/-* BmMSCs were not statistically significant, whereas it was statistically significant for alkaline phosphatase, osterix, osteonectin, and osteocalcin at later timepoints in the *Grk3-*deficient shRNA knockdown cultures. We hypothesize that this may be due to earlier passages that were available for testing the *Grk3-*KD cultures compared to later passages that were available for the *Grk3-/-* BmMSCs.

*Grk3-/-* BmMSC cultures had higher levels of CXCL12 at baseline upon equal seeding densities, and one interpretation is that *Grk3-/-* BmMSCs could have an enhanced CXCL12 secretory function, such as has been described of CXCL12-abundant reticular stromal cells in the bone marrow [22]. However, our study also detected enhanced proliferation of *Grk3-/-* BmMSCs (Fig. 3B), and the CXCL12 per cell calculation (Fig. 3C) does not support increased secretory function. This suggests the levels of CXCL12 detection at most timepoints are likely due to the increased number of *Grk3-/-* BmMSCs in culture.

We hypothesize that these in vitro findings may additionally contribute toward mechanisms underlying the enhanced hematopoiesis phenotype that we observed in the *Grk3-/-* mouse [4] and appear to be less relevant in osteoporosis or mature bone development (Fig. 4). Previous studies have demonstrated depletion of CXCL12 or total numbers of mesenchymal stem cells decrease the bone marrow hematopoietic stem cell pool and repopulating activity [22–24]. Thus, we might conclude the enhanced numbers of HSPCs observed in the *Grk3-/-* mouse bone marrow [4] could be attributed at least in part to the *Grk3-/-* BmMSC-enhanced phenotypes described here. However, we recognize that selective *Grk3* gene targeting of mesenchymal stem cells and/or osteoblasts in vivo would be necessary to definitively conclude this – the timing of which may prove challenging in targeting which stage of stem cell development is most relevant for *Grk3* expression. These are the goals of future studies, as opposed to the focus of this initial characterization of *Grk3-/-* BmMSCs and identification of candidate regulatory GPCRs.

Neither young (8–12-week-old) nor aged (17–20-month-old) *Grk3-/-* mice show enhancement of mature trabecular bone formation in vivo (Fig. 4A-D). Possible explanations of this finding may be attributed to our *Grk3-/-* conventional knockout mouse model, where there may be other compensatory cellular interactions at play. Importantly, *Grk3* expression levels have been found to be either undetectable or minimally expressed in osteoblastic cell lines [45]. This physiologic downregulation of *Grk3* in more mature osteoblasts, as opposed to precursor stem cells, could also explain why differences were not observed in trabecular bone in vivo.

To test whether GRK3 may regulate S1P-stimulated S1PR, our studies utilized SKI to efficiently reduce the active S1P ligand in extended time-course studies and thereby block ligand activation for all S1PR family members expressed on BmMSCs (Fig. 5 and Additional File 5: Fig. S5A and 5B). These results did show that the enhanced proliferation and osteogenic differentiation of *Grk3-/-* BmMSCs can be reduced to WT levels with effective removal of the S1PR ligand. Of note, we also tested specific S1PR antagonism targeting S1PR1 and S1PR3 [46]. However, we observed BmMSC cellular toxicity in our extended time-course studies while using similar concentrations of vehicle (DMSO) alone that was present in the solubilized S1PR1 and S1PR3 antagonist (VPC23019, Cayman Chemical) group, making this data of receptor-specific antagonism not interpretable. Consequently, our culture studies with BmMSCs and S1PR antagonism could not identify which S1PR subtype expressed on BmMSCs elicit the enhanced functionality due to concerns about cell viability and experimental rigor.

GRK3 is a ubiquitously expressed kinase that regulates a number of GPCRs, including parathyroid hormone receptor (PTH-R) [47]. Although our cellular characterization studies suggest loss of GRK3 function affects S1PR signaling leading to enhanced BmMSC functionality ex vivo, we considered GRK3 regulation of parathyroid hormone/parathyroid hormone receptor (PTH/PTH-R), since PTH/PTH-R has been linked to osteoblast differentiation and enhanced hematopoiesis [48, 49]. However, ligand PTH is not readily available in ex vivo cultures, and PTH-R was absent from a RNA-seq database evaluation of readily expressed GPCRs on (human) BmMSCs (ENCODE GEO accession: GSE90273) [50]. Alternatively, the RNA-seq database showed S1PR expression on BmMSC, and it is well established that S1P is readily available in horse and fetal bovine serum (i.e., BmMSC culture media) [31].

Contrary to our finding, Hosogane et al. proposed CXCL12 regulates bone morphogenetic protein 2-induced osteogenic differentiation of primary mesenchymal stem cells demonstrated in part by treatment with AMD3100 (CXCR4 antagonist) to show reduction of osteogenic differentiation [51]. Our functional tests with CXCL12 stimulation or CXCR4 signaling inhibition with AMD3100 resulted in no effect (Additional File 3: Fig. S3). One explanation for differences between our data is that CXCR4 is detected on human BmMSCs [52, 53] but less so on murine BmMSCs cultured in vitro [54]. Other possible explanations for outcome differences may be that the Hosogane et al. MSC cultures were different based on isolation technique, cell surface marker expression, differences in AMD3100 concentration, or a combination of these variables.

To investigate whether the enhanced functionalities of *Grk3-/-* BmMSCs were due to changes in S1PR signaling, we tested the ERK signaling pathway since previous studies suggest ERK activation promotes both mesenchymal stem cell [33] and pre-osteoblastic cell line [34] osteogenic differentiation, as well as mesenchymal stem cell proliferation [12]. Our previous work has shown GRK deficiency elicits sustained ERK signaling due to prolonged GPCR surface expression and failure to desensitize ligand-GPCR stimulation [4]; therefore, we hypothesized loss of GRK3 in BmMSCs may elicit sustained ERK activation through S1P/S1PR ligation. Our results show stimulation with S1P prolongs *Grk3-/-* BmMSC ERK1/2 signaling (Fig. 6) and thus suggest S1P-ligand-induced signaling of *Grk3-/-* BmMSCs may promote enhanced cell functions mediated through the ERK1/2 signaling pathway.

**Fig. 8:**
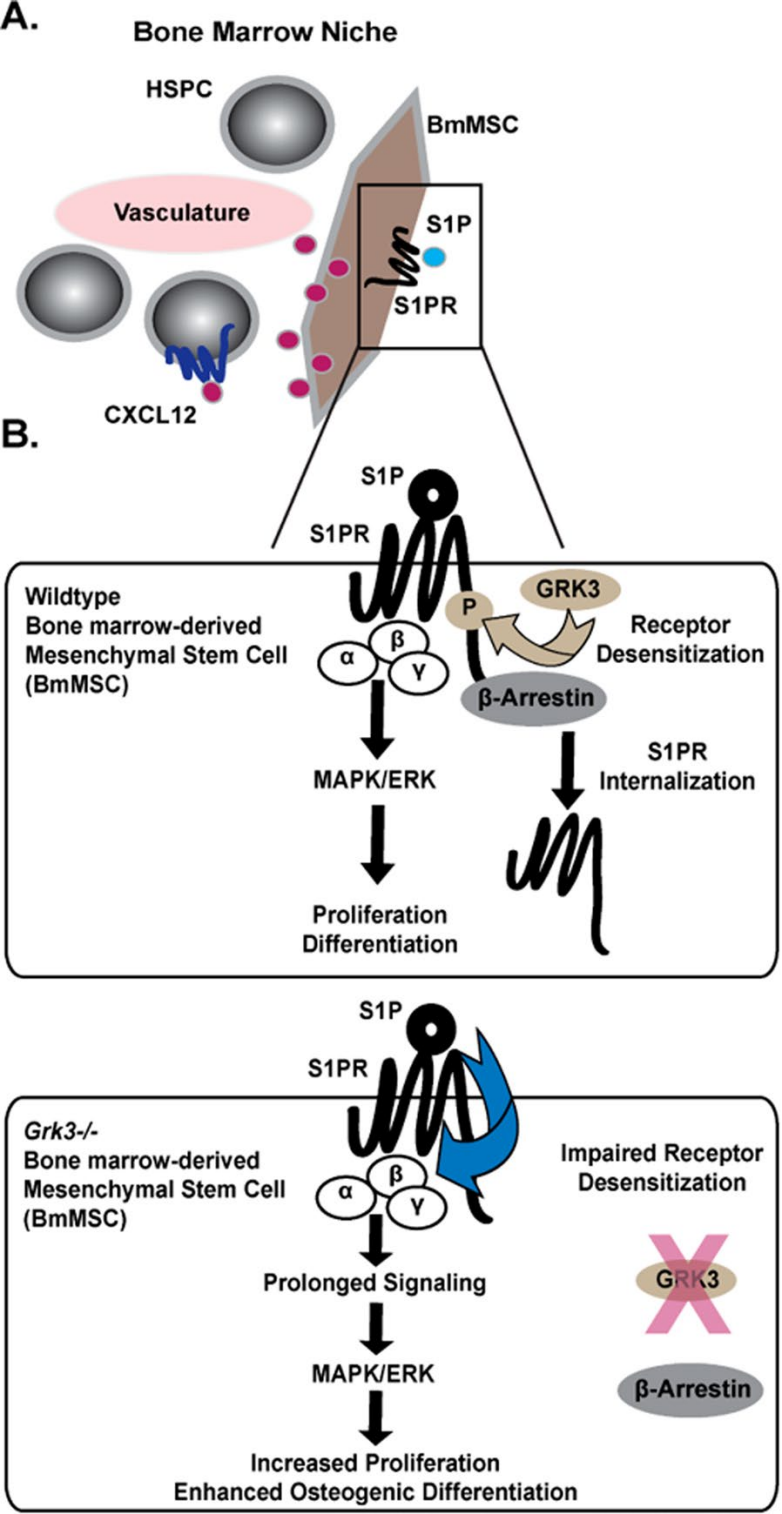
Proposed model for GRK3-deficient BmMSCs within the niche. (**A**) Our studies suggest BmMSCs deficient in GRK3 secrete more CXCL12 and proliferate more rapidly, which may increase support to hematopoietic stem and progenitor cell (HSPC) development. (**B**, top) We propose GRK3 desensitizes S1PR and recruits β-arrestin to affect ERK1/2 signaling and receptor internalization. (**B**, bottom) *Grk3-/-* BmMSCs have enhanced functions of proliferation and osteogenic differentiation, and this may be mediated through decreased S1PR1 internalization (i.e., more surface expression) and prolonged late-phase ERK1/2 activation

S1PR1 and S1PR3 have been shown to stimulate BmMSC function [36], but S1PR1-mediated enhanced proliferation has been demonstrated in adipose-derived MSCs [12]. Thus, we focused our receptor signaling regulation studies on S1PR1 to determine whether GRK3 enhances recruitment of β-arrestin-2, a protein necessary for GPCR internalization and another pathway of GPCR regulation in addition to receptor desensitization by GRKs. Previous studies have shown GRK2, which shares high homology with GRK3, phosphorylates the C-terminus of S1PR1 [55, 56], and activated S1PR1 recruits β-arrestin-2 for receptor internalization [56, 57]. Furthermore, cells deficient in GRK2 have high levels of surface S1PR1 [39]. Consequently, we compared GRK2 as a positive control for our modified-TANGO assay. We demonstrate GRK3 enhances β-arrestin-2 recruitment to the C-terminus of S1PR1, as does GRK2 (Fig. 7A). Furthermore, BmMSCs deficient in GRK3 had decreased S1PR1 internalization (i.e., more surface expression) (Fig. 7B). Thus, we propose loss of GRK3 affects S1PR1 regulation on the BmMSC by impairing S1PR desensitization, prolonging S1PR internalization leading to enhanced surface receptor expression and ligand activation with S1P that in turn sustains late-phase ERK1/2 activation (Fig. 8).

These data show increased MSC osteogenic potential and proliferative growth by targeting the GRK3-mediated GPCR signaling pathway S1P/S1PR. Because the GPCR superfamily has been one of the more successfully targeted class of receptors for drug therapy [58], GPCR antagonism of a receptor that GRK3 regulates or direct kinase inhibition of GRK3 is readily achievable. Indeed, several antagonists of the S1PR signaling pathway are under study for preclinical and clinical use [2]. Thus, future directions in GRK3 signaling in stem cell and bone biology may provide mechanistic insight and therapeutic advancement for several clinical diseases and treatment indications, including but not limited to metabolic bone disease, stem cell transplantation, healing of fracture, and/or in vivo scaffolding.

## Conclusion

In summary, our study reveals how *Grk3-/-* BmMSCs have enhanced functions of osteogenic differentiation and proliferation that is attributed to altered S1P/S1PR (Fig. 7A). We demonstrate GRK3 recruits β-arrestin-2, a protein necessary for receptor internalization, to the C-terminus of S1PR1, and BmMSCs lacking GRK3 regulation have impaired S1PR1 internalization and prolonged ERK1/2 signaling (Fig. 7B). Our work suggests GRK3 regulates S1PR on BmMSCs, and we propose lack of such regulation affects BmMSC functionality. This research could have future implications for cellular-based treatment of cancer, autoimmunity, or fracture.

## Supplementary Information

**Additional file 1:** Fig. S1. Mouse mesenchymal stem cell marker panel. Representative WT (top) and *Grk3-/-* (bottom) BmMSCs expression of positive markers CD106, CD73, CD44, CD29, Sca1 and negative hematopoietic markers CD45 and CD11b (macrophage). Data plotted as percent (normalized to mode)

**Additional file 2:** Fig. S2. CD73 expression on BmMSCs. WT (*n* = 4) and *Grk3-/-* (*n* = 4) BmMSCs expression of positive CD73 for passages 8-11 of 4 independent isolated batches for each genotype

**Additional file 3: **Fig. S3. BmMSC proliferation was not affected by CXCL12 stimulation or CXCR4 signaling inhibition with AMD3100. (**A**) Cellular proliferation was not enhanced in the presence of CXCL12, a CXCR4 agonist, at various concentrations, and (**B**) cellular proliferation was not reduced in the presence of AMD3100, a CXCR4 antagonist at various concentrations. Data represent mean ± SEM, *n* = 3

**Additional file 4:** Fig. S4. WT and *Grk3-/-* BmMSCs have comparable sphingosine kinase activity WT and *Grk3-/-* BmMSCs were treated with fluorescein-labeled sphingosine to assess the BmMSC ability to convert sphingosine to active ligand sphingosine-1-phosphate (S1P) through sphingosine kinase (SphK) activity. WT and *Grk3-/-* BmMSC converted sphingosine into S1P (i.e., SphK activity) at comparable levels. Data represent mean ± SEM, *n* = 3

**Additional file 5:** Fig. S5. WT and *Grk3-/-* BmMSCs express S1PR1. (**A**) Immunoblot detection of S1PR1 from both WT and *Grk3-/-* BmMSC lysates, and (**B**) flow cytometry detection of surface S1PR1 (PE-conjugated) on Sca1+ (APC-conjugated) WT and *Grk3-/-* BmMSCs, passages 11, 12

## Data Availability

The data obtained in these studies is contained within the manuscript and Additional files 1, 2, 3, 4, and 5.

## Abbreviations

ALP: Alkaline phosphatase
BmMSC: Bone marrow-derived mesenchymal stem cell
CCK-8: Cell counting kit-8
CEM: Complete expansion media
CIM: Complete isolation media
CXCL12: C-X-C motif chemokine ligand 12
CXCR4: C-X-C chemokine receptor type 4
DMSO: Dimethyl sulfoxide
ERK: Extracellular-related kinase
FBS: Fetal bovine serum
GRK2: G protein-coupled receptor kinase 2
GRK3: G protein-coupled receptor kinase 3
GRK3-KD: G protein-coupled receptor kinase 3 knock-down
GPCR: G protein-coupled receptor
HS: Horse serum
HSPC: Hematopoietic stem and progenitor cell
IMDM: Iscove's modified Dulbecco's medium
LSK: Lin(-) Sca-1(+) c-Kit(+) hematopoietic stem and progenitor cells
PTH: Parathyroid hormone
PTH-R: Parathyroid hormone receptor
qRT-PCR: Quantitative reverse transcription polymerase chain reaction
S1P: Sphingosine-1-phosphate
S1PR: Sphingosine-1-phosphate receptor
SKI: Sphingosine kinase inhibitor
WT: Wild type
µCT: Microcomputed tomography
