# Supplementary figures and images for: G protein-coupled receptor kinase 3 modulates mesenchymal stem cell proliferation and differentiation through sphingosine-1-phosphate receptor regulation
Source: Stem Cell Res Ther. 2022 Jan 29;13:37. doi: 10.1186/s13287-022-02715-4 (PMC8800243; doi:10.1186/s13287-022-02715-4)

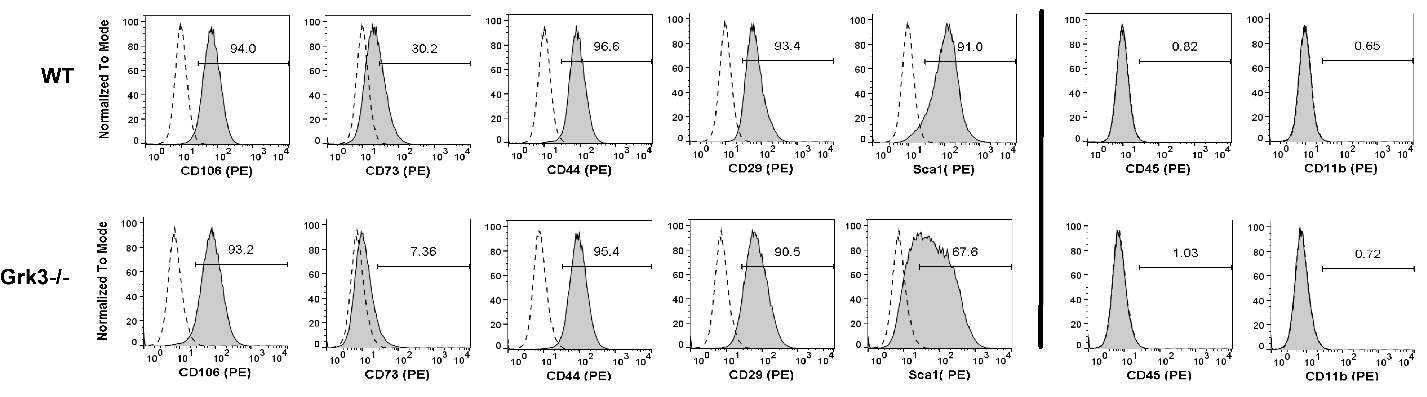

Supplement: Supplementary file 1 — Additional file 1: Fig. S1. Mouse mesenchymal stem cell marker panel. Representative WT (top) and Grk3-/- (bottom) BmMSCs expression of positive markers CD106, CD73, CD44, CD29, Sca1 and negative hematopoietic markers CD45 and CD11b (macrophage). Data plotted as percent (normalized to mode) [file 13287_2022_2715_MOESM1_ESM.tif]

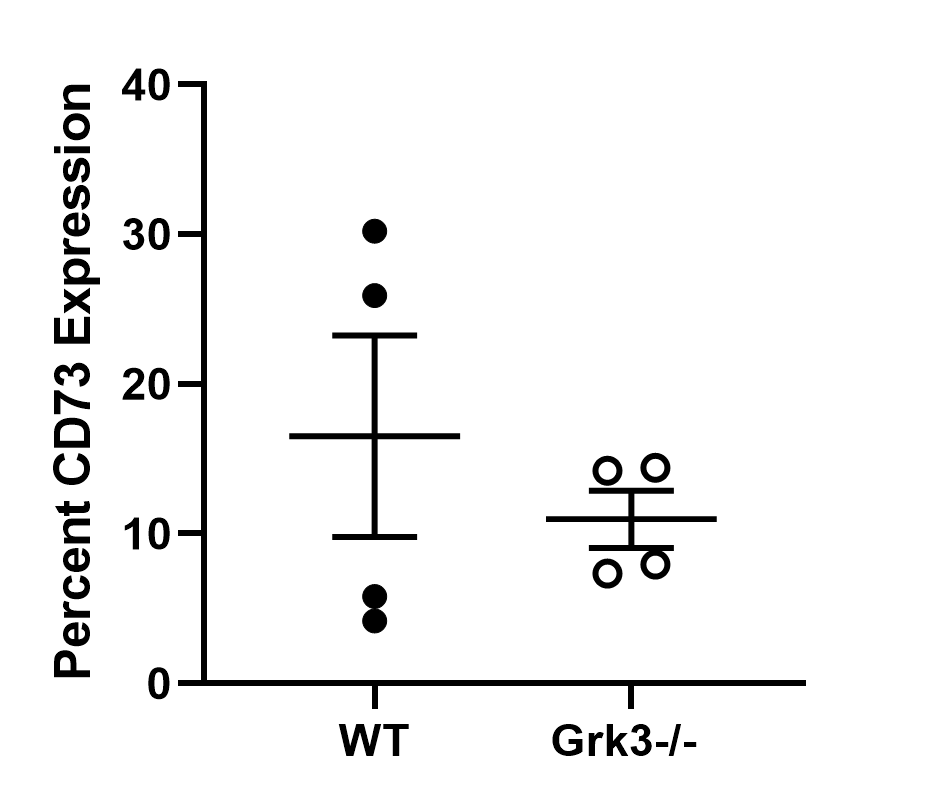

Supplement: Supplementary file 2 — Additional file 2: Fig. S2. CD73 expression on BmMSCs. WT (n = 4) and Grk3-/- (n = 4) BmMSCs expression of positive CD73 for passages 8-11 of 4 independent isolated batches for each genotype [file 13287_2022_2715_MOESM2_ESM.tif]

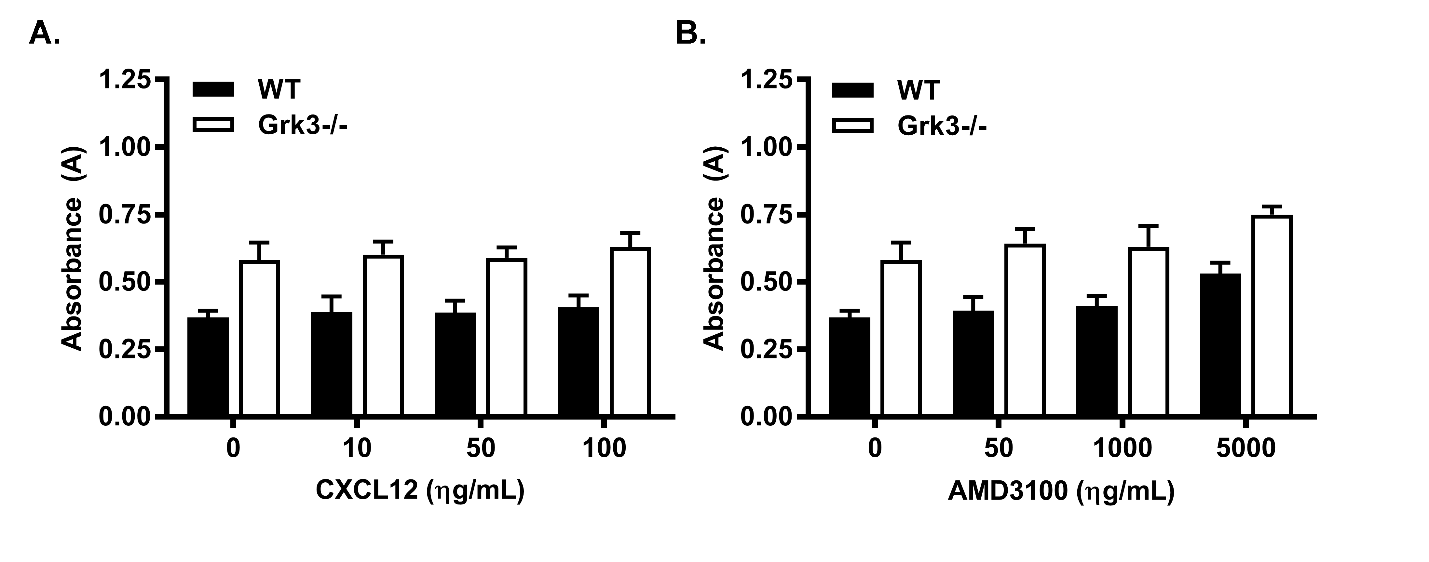

Supplement: Supplementary file 3 — Additional file 3: Fig. S3. BmMSC proliferation was not affected by CXCL12 stimulation or CXCR4 signaling inhibition with AMD3100. (A) Cellular proliferation was not enhanced in the presence of CXCL12, a CXCR4 agonist, at various concentrations, and (B) cellular proliferation was not reduced in the presence of AMD3100, a CXCR4 antagonist at various concentrations. Data represent mean ± SEM, n = 3 [file 13287_2022_2715_MOESM3_ESM.tif]

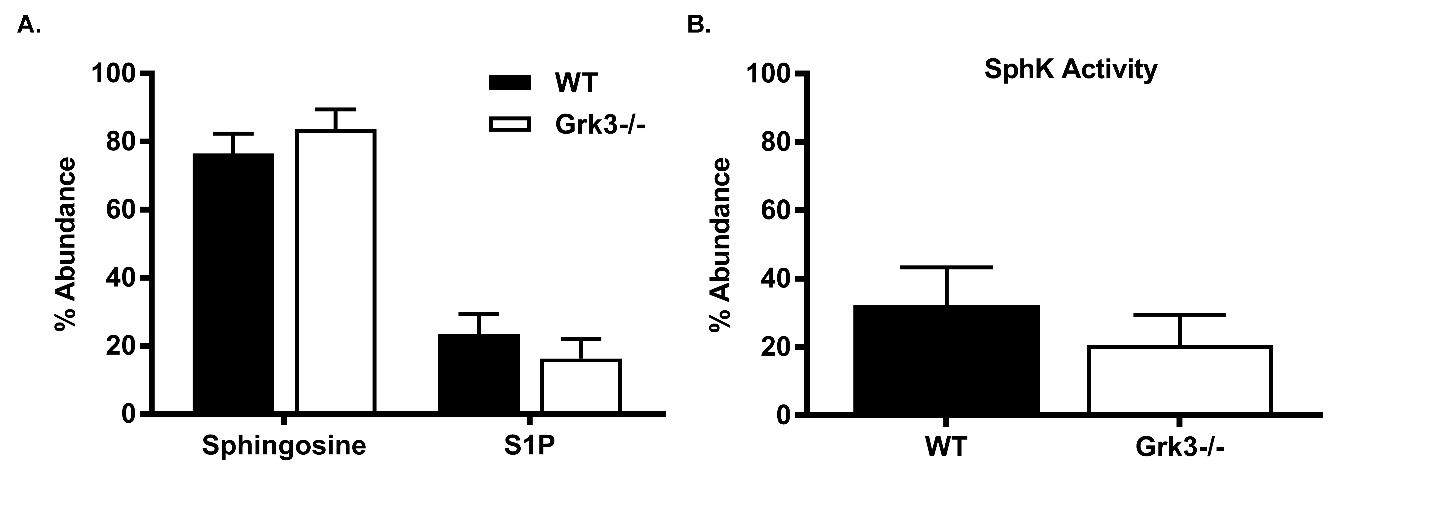

Supplement: Supplementary file 4 — Additional file 4: Fig. S4. WT and Grk3-/- BmMSCs have comparable sphingosine kinase activity WT and Grk3-/- BmMSCs were treated with fluorescein-labeled sphingosine to assess the BmMSC ability to convert sphingosine to active ligand sphingosine-1-phosphate (S1P) through sphingosine kinase (SphK) activity. WT and Grk3-/- BmMSC converted sphingosine into S1P (i.e., SphK activity) at comparable levels. Data represent mean ± SEM, n = 3 [file 13287_2022_2715_MOESM4_ESM.tif]

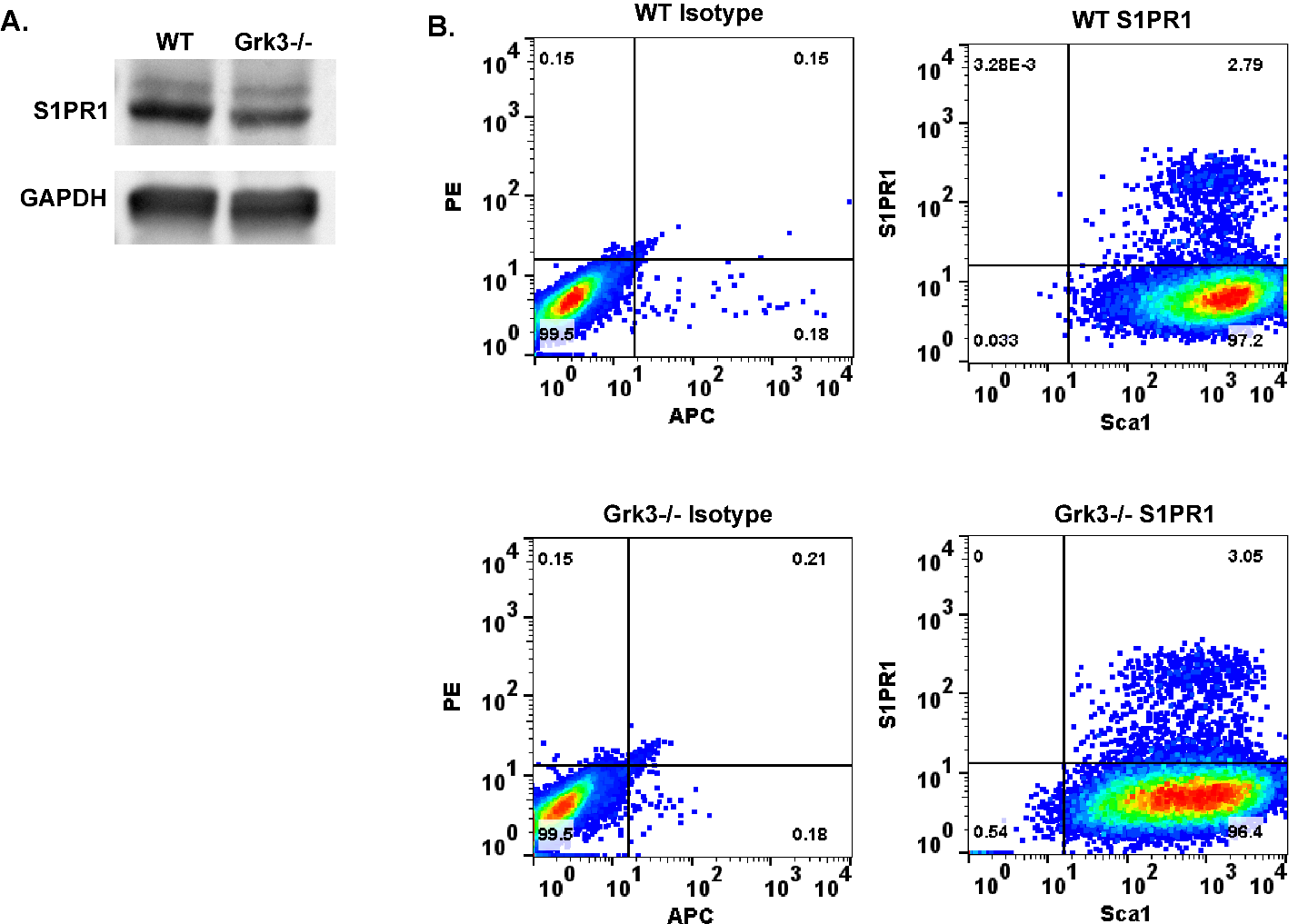

Supplement: Supplementary file 5 — Additional file 5: Fig. S5. WT and Grk3-/- BmMSCs express S1PR1. (A) Immunoblot detection of S1PR1 from both WT and Grk3-/- BmMSC lysates, and (B) flow cytometry detection of surface S1PR1 (PE-conjugated) on Sca1+ (APC-conjugated) WT and Grk3-/- BmMSCs, passages 11, 12 [file 13287_2022_2715_MOESM5_ESM.tif]
